# Acute kidney injury associated with *Plasmodium malariae* infection

**DOI:** 10.1186/1475-2875-13-226

**Published:** 2014-06-07

**Authors:** Aida S Badiane, Khadim Diongue, Seydou Diallo, Aliou A Ndongo, Cyrille K Diedhiou, Awa B Deme, Diallo Ma, Mouhamadou Ndiaye, Mame C Seck, Therese Dieng, Omar Ndir, Souleymane Mboup, Daouda Ndiaye

**Affiliations:** 1 Laboratory of Parasitology-Mycology LeDantec Hospital, 30 Pasteur Avenue, Dakar, Senegal; 2 Laboratory of Bacteriology-Virology, Malaria Unit, LeDantec Hospital, 30 Avenue Pasteur, Fann, Dakar, Senegal; 3 Internal Medicine, Laveran, LeDantec Hospital, 30 Avenue Pasteur, Dakar, Senegal; 4 Laboratory of Parasitology-Mycology Cheikh Anta Diop University of Dakar, Avenue Cheikh Anta Diop Dakar, BP 5005 Fann, Dakar, Senegal

**Keywords:** Plasmodium malariae, Kidney injury, Senegal

## Abstract

According to current estimates, *Plasmodium malariae* is not very common in Senegal, as more than 98% of malaria cases are suspected to be due to *Plasmodium falciparum*. However, it is possible that other malarial species are being under-reported or misdiagnosed. This is a report of a case of *P. malariae* in a 30-year-old man previously hospitalized with acute kidney injury after treatment with quinine and re-hospitalized three months later. He was diagnosed with renal cortical necrosis post malaria treatment. *Plasmodium malariae* was identified with light microscope and confirmed using species-specific small-subunit rRNA (ssrRNA) amplification.

The patient was treated for malaria with intravenous quinine for seven days, followed by three days of oral treatment; the bacterial infection was treated using ceftriaxone during the first hospitalization and ciprofloxacin associated with ceftriaxone the second time. He also had four rounds of dialysis after which he partially recovered the renal function. Given the complications that can be caused by *P. malariae* infection, it should be systematically looked for, even if the predominant species is *P. falciparum* in Senegal.

## Background

Senegal is a malaria endemic region with a predominance of *Plasmodium falciparum*. Over the few last years, the malaria burden in Senegal has declined but the disease remains endemic. Other species such as *Plasmodium malariae*, and *Plasmodium ovale* are present in Senegal, but recent data on their frequencies is lacking. Although, infections with non-*falciparum* species generally result in mild disease [1,2], their identification is important. In fact, *P. malariae* is known not to cause severe symptoms, but is sometimes associated with renal pathology [3-6]. Evidence suggests that *P. malariae* does not cause relapse, but low level parasitaemia can persist for years and recrudescent infections are well documented [7] and can cause disease years after the initial infection [8]. As falciparum malaria can cause severe and fatal disease, significant research has focused on improving its diagnosis and treatment. This has contributed to less research focus on the other human malarias and their disease burden being underestimated. This is a report of a case of *P. malariae* diagnosed in a patient living in Dakar and previously hospitalized with acute kidney injury.

## Case presentation

A 30-year-old man first presented at a health centre in Dakar, Senegal (Abdoul Aziz Sy health care Centre) with fever, headaches, myalgia, and vomiting. He was hospitalized and treated for malaria with quinine, based on clinical diagnosis although malaria diagnosis was not confirmed by biological test. The patient failed to recover and he was referred to the Infectious Disease Clinic (Fann Hospital, Dakar, a national reference hospital) where he was treated with furosemide, metronidazole, metopimazine, dycinone and omeprazole, he presented with anuria for three days. He was negative for malaria by microscopy at Fann Hospital. He was transferred to the Nephrology Clinic (Aristide LeDantec Hospital, a national reference hospital) with creatinine of 152.52 mg/L and urea of 2.52 g/L. At admission, he received a first round of dialysis at Aristide LeDantec Hospital, where the molecular diagnostic unit is located.

The patient presented fever, anaemia, ascitis of low abundance, insomnia and asthenia. He received ceftriaxone 1 g/day, and the furosemide continued to be administered. During the course of hospitalization, he received four rounds of dialysis and three blood transfusions. Creatinine, urea and potassium were measured to monitor the renal function during hospitalization (Table 1). At release his creatinine was 82.69 mg/L and urea was 1.62 g/L (Table 1).

**Table 1:** Biological follow up of the renal function

	**Creatinine (mg/L)**	**Urea (g/L)**	**Potassium (mmol/L)**	**Dialysis**
7/11/12 (Jo)	124.52	2.52	4.7	None
10/11/12 (J3)			5.3	Yes
12/11/12/ (J5)	166.25	3.13	4.7	Yes
15/11/12 (J8)	161.47	2.62		None
20/11/12/ (J13)	217	4.31		None
21/11/12 (J14)				Yes
22/11/12 (J15)	100	1.49		None
23/11/12 (J16)	115.55	1.75		None
24/11/12 (J17)				Yes
27/11/12 (J20)	87.89	1.89		None
3/12/12	82	1.62		None

The patient was hospitalized again three months later in another health centre (Philippe Senghor health care centre); suffering from diarrhoea, fever, chills, asthenia and anorexia for a week. Given his renal condition, he was referred to Aristide LeDantec Hospital again, with creatinine of 67 mg/L and urea of 2.40 g/L. The potassium was 6.4 mmol/L; therefore, a dialysis was performed in emergency. After a month of treatment the laboratory test results were creatinine, 34.5 mg/L; creatinine clearance, 27.7 ml/min; sodium (Na^+^), 132 mmol/L; potassium (K^+^), 5.1 mmol/L; chloride (Cl-), 108 mmol/L; total protein, 71.3 g/L.

He was treated with ceftriaxone 1 g per day and ciprofloxacin 1 g per day because typhoid was suspected and the doses were adapted to his renal condition. But a non-fermentative Gram negative bacillus was isolated from blood culture and typhoid investigation by Widal test and stool culture was negative. Malaria diagnosis by microscopy was performed one week after his admission and was positive for *P. malariae.* He was then treated with quinine 500 mg intravenous (1/2 ampoule in 250 mm^3^ of 10% serum glucose for 4 hours) during 7 days, then by oral route for three days and paracetamol intramuscular was administered for the pain. Renal biopsy was prescribed and showed parenchyme fragments with 80% nephrotic reduction. Secondary focal segmental glomerulosclerosis (FSGS) lesions were seen for 30% of residual glomeruli, and an extended mutilating interstitial fibrosis and atherosclerosis lesions of variable distribution between severe to moderate was also noted.

The appearance evoked a subtotal cortical necrosis probably on severe nephroangiosclerosis lesions and pyelonephritis was probably associated. The diagnosis of cortical necrosis post malaria was, therefore, established.

At the end of hospitalization, creatinine was 29.4 mg/L, urea 0.48 g/L and the glomerular filtration rate (GFR) 30 ml/min, which means the renal function was not fully recovered and is in agreement with the renal necrosis. The patient is still being monitored in the nephrology clinic.

### Light microscopy results

**Figure 1:**
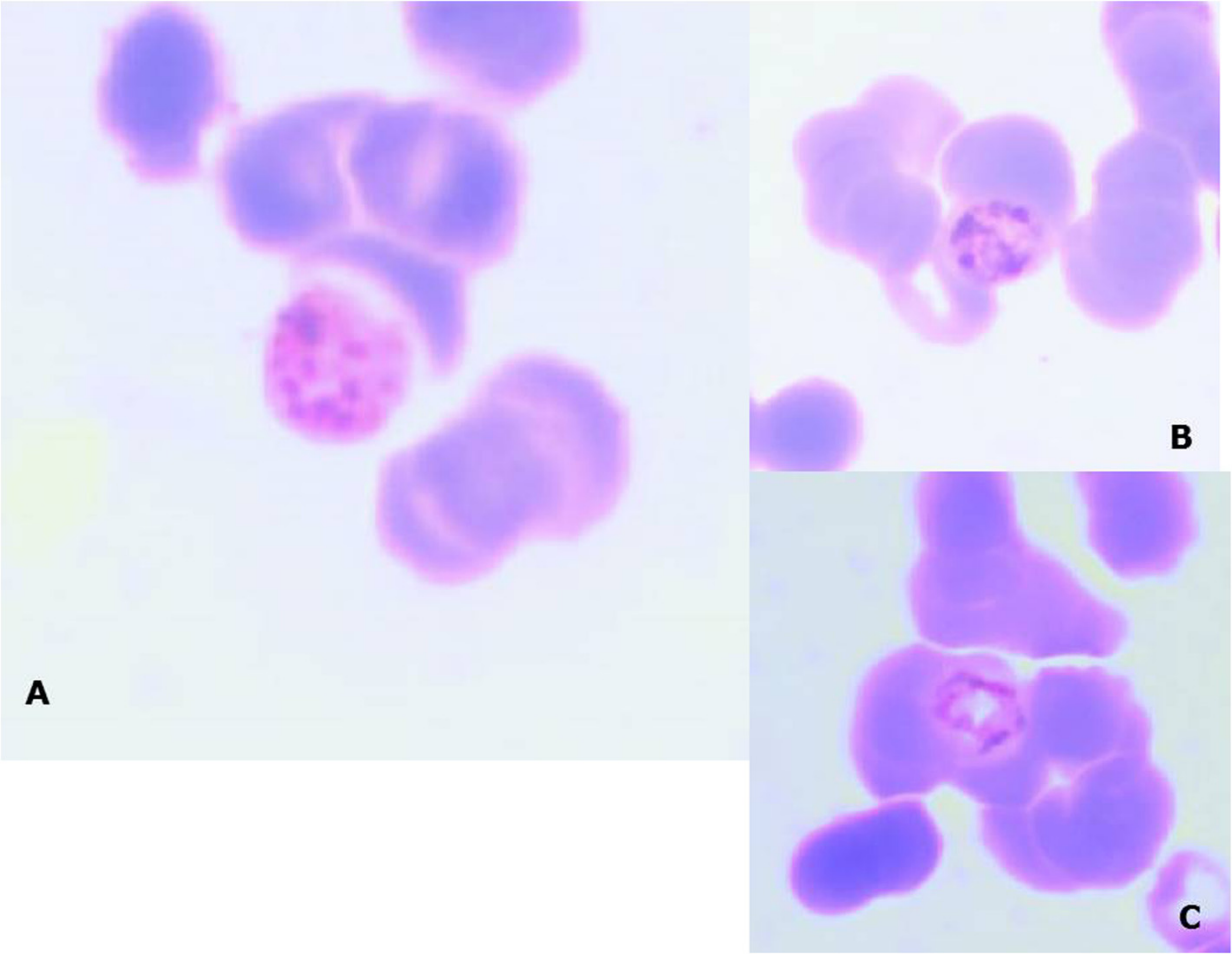
**Giemsa-stained thin smear of patient’s peripheral blood. A**: Schizont of *P. malariae* containing merozoites (6 to 12) with large nuclei and the rosette pattern. **B** and **C**: Trophozoites of *P. malariae* “Basket-form” in a thin smear.

Thin and thick blood smears were sent to the laboratory of parasitology at Le Dantec Hospital for malaria diagnosis by light microscopy. Slides were stained with 10% Giemsa for 15 minutes. *Plasmodium malariae* was diagnosed with a parasite density of 18.402 trophozoites/μl (Figure 1).

### Rapid diagnosis test results

To confirm, the microscopy result, we asked for a blood sample and performed the RDT/HRP2 SD Bioline Malaria Ag Pf with a (sensitivity of 99.7% for 100 parasites/μl and a specificity of 99.5%), which is specific to *P. falciparum.* The test was negative ruling out *P. falciparum* infection, which is more common in Senegal.

### Molecular diagnostic testing ssrRNA gene amplification by polymerase chain reaction (PCR)

To confirm the diagnosis of *P. malariae*, DNA was extracted from whole blood with the QIAamp DNA blood mini kit (Qiagen). The ssrRNA gene was amplified as previously described [9,10] with minor modifications. First round PCR to detect *Plasmodium* genus was performed using the following primers rPLUf 5’- TTA AAA TTG TTG CAG TTA AAA CG and rPLUr 5’- CCT GTT GTT GCC TTA AAC TTC. The second round PCR with primers specific to 4 *Plasmodium* species:

*P. malariae* rMALf5’- ATA ACA TAG TTG TAC GTT AAG AAT AAC CGC and rMALr AAA ATT CCC ATG CAT AAA AAA TTA TAC AAA

*P. falciparum* rFALf 5’- TTA AAC TGG TTT GGG AAA ACC AAA TAT ATT and rFALr 5’- ACA CAA TGA ACT CAA TCA TGA CTA CCC GTC

*P. ovale* rOVAf 5’- ATC TCT TTT GCT ATT TTT TAG TAT TGG AGA and rOVAr 5’- GGA AAA GGA CAC ATT AAT TGT ATC CTA GTG

*P. vivax* rVIVf 5’- CGC TTC TAG CTT AAT CCA CAT AAC TGA TAC and rVIVr 5’- ACT TCC AAG CCG AAG CAA AGA AAG TCC TTA

PCR reactions were performed in a final volume of 20 μl: GoTaq (Promega Green Master Mix 2X) 6 μl, forward primer 10 pmol/μl (1 μl), reverse primer 10 pmol/μl (1 μl), DNA 2 μl, and 10 μl of distilled water.

The PCR programme was as follows: initial denaturation at 94°C for 4 min, then 35 cycles of 94 for 30 sec, 55°C for 1 min (first round annealing) or 58°C for 1 min (second round annealing), 72°C for 1 min, final extension at 72°C for 4 min, and 4°C at the end.

**Figure 2:**
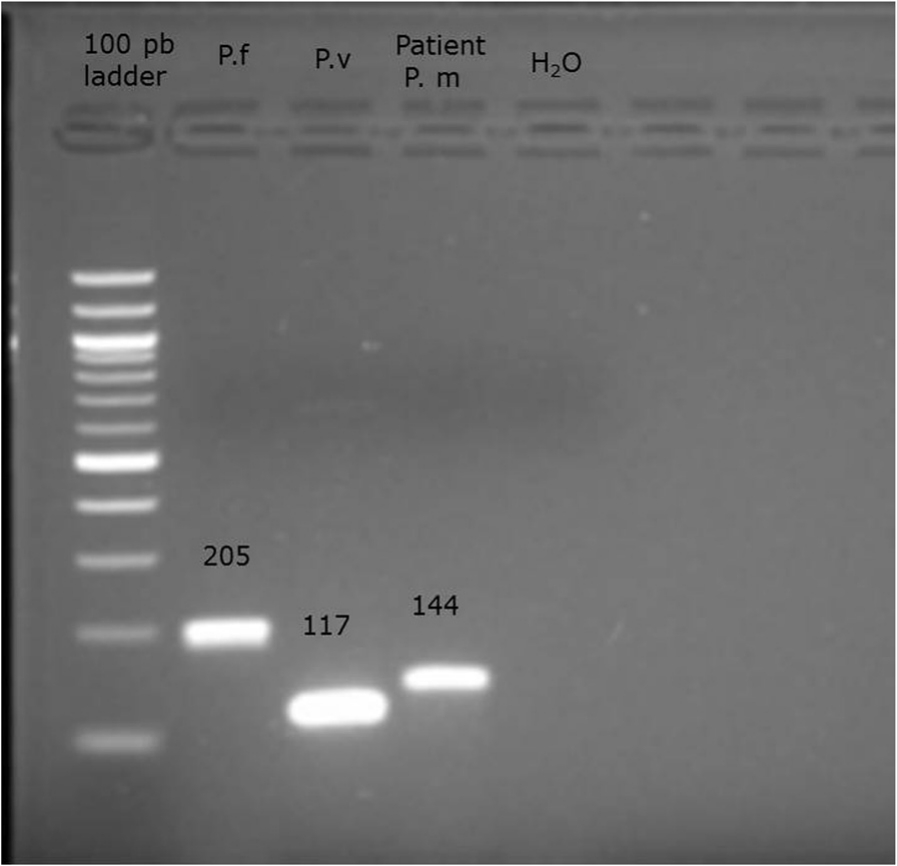
**PCR results for *Plasmodium *species-specific 18S rRNA.** 144-bp fragment corresponds to *P. malariae* was detected from the PCR-amplified product of thev patient’s blood 205 bp fragment corresponds to *P. falciparum* used as a positive control 117 pb fragment corresponds to *P. vivax* used as positive control. Ladder: DNA size marker (100 pb).

Samples were run on an agarose gel and species determined based on product size [9]. *Plasmodium malariae* was diagnosed at the size band of 144 base pairs (bp) (Figure 2). *Plasmodium falciparum* and *Plasmodium vivax* strains available in the laboratory, were used as positive controls.

## Discussion

Although *P. malariae* does not cause life-threatening infection, it has been associated with kidney injury [11-15], which is a very serious condition. Unlike *P. falciparum*, *P. malariae* infection is chronic and parasitaemia is often low which makes microscopic diagnosis difficult. In Senegal, most rapid diagnosis tests (RDTs) used in public settings only detect the Histidine Rich Protein 2 (HRP2) of *P. falciparum* and PCR is primarily performed for research purposes. Therefore, patients presenting with *P. malariae* can easily misdiagnosed and are often treated for bacterial infections.

The recommendation of the National Malaria Control Programme is to combine RDTs with the gold-standard microscopy in endemic areas; however, sometimes only RDTs are used, often in rural areas where the expertise and the equipment for performing microscopy are lacking. RDTs were developed particularly for use in low resource settings to facilitate the diagnosis of malaria in the absence of microscopy. Light microscopy can detect all *Plasmodium* species but requires extensive training and specialized expertise. Different types of RDTs exist and the choice of RDT implemented depends on the species that circulate in the area [16]. The most common *Plasmodium* species causing clinical malaria in Senegal is *P. falciparum,* which represents more than 98% cases. While other species are present, they are predicted to be at much lower frequencies. Additionally, co-infections occurring in *P. falciparum* endemic areas are often difficult to detect by microscopy, in a setting of high *falciparum* parasitaemia. The low parasite density of *P. malariae* compared to *P. falciparum* could partially explain why it is under-diagnosed even by light microscopy [17,18].

Although *P. malariae* is not known to cause relapse, infected red blood cells can persist for long periods of time at low densities (<500 parasites/μl). These low densities are due to the fewer merozoites released after schizont rupture, increased erythrocytic cycle duration (72 hours versus 48 hours for *P. falciparum*), erythrocyte preference for invasion (mature red blood cells) and the host factors such as immunity. One study reports *P. malariae* parasites isolated from a patient infected 50years earlier while in China [19].

Although not a common disease in Senegal, *P. malariae* can cause public health problems as it is associated with kidney injury which can be complicated and costly to treat in low income countries. Given the complications, it is time to re-evaluate the current diagnostic of choice, in order to prevent such pathologies associated with *P. malariae* infection.

## Conclusion

For this case the first diagnosis of malaria was only based on the clinic because biological tests were not available when the patient came to the hospital. During his first hospitalization he has been treated for malaria with quinine, after which he developed kidney injury but whether quinine or the *Plasmodium* infection was responsible of the injury remains unclear because there is not enough evidence to establish the aetiology.

### Consent

Written informed consent was obtained from the patient for publication of this Case report and any accompanying images. A copy of the written consent is available for review by the Editor-in-Chief of this journal.

## Abbreviations

ssrRNA: Small-subunit rRNA; FSGS: Focal segmental glomerulosclerosis; GFR: Glomerular filtration rate; RDT: Rapid diagnosis test; HRP2: Histidine rich protein-2; Bp: Base pairs.

## Competing interests

Authors declare that they have no any competing interests.

## Authors’ contributions

ASB confirmed and interpreted the biological diagnosis and wrote the manuscript. KD and SD made the microscopic diagnosis. AAN gave the clinical examination. CKD and ABD made the molecular diagnosis. MN, MCS, TD, ON helped for the literature and biological diagnosis. SM and DN gave constructive advice and reviewed the manuscript. All authors have read and approved the final version of manuscript.
